# The trends and outcomes of inflammatory bowel disease surgery during the COVID‐19 pandemic: A retrospective propensity score‐matched analysis from a multi‐institutional research network

**DOI:** 10.1002/hsr2.70107

**Published:** 2024-09-29

**Authors:** Fiona Wu, Gema H. Ibarburu, Caris Grimes

**Affiliations:** ^1^ Department of General Surgery, East Sussex Healthcare NHS Trust Conquest Hospital, The Ridge Hastings UK; ^2^ TriNetX Parque Empresarial Prado del Espino Madrid Spain; ^3^ King's College London, Strand London UK

**Keywords:** COVID, inflammatory bowel disease (IBD), outcomes, pandemic, trends

## Abstract

**Background and Aims:**

The coronavirus disease 2019 (COVID‐19) pandemic has affected the management of inflammatory bowel disease (IBD) patients. Elective operations and surveillance endoscopies were postponed for IBD patients to preserve healthcare resources and to prevent the spread of COVID‐19. This study aimed to describe the trends and outcomes of IBD surgery during the pandemic.

**Methods:**

This was a retrospective propensity score‐matched analysis using data extracted from TriNetX, a multi‐institutional research database. IBD patients admitted for surgery were identified between March 2019 to February 2020 (prepandemic) and March 2020 to February 2023 (pandemic). The monthly volume of IBD surgical procedures was compared during the pandemic to the prepandemic period. After matching, the risk of adverse outcomes following IBD surgery was compared between the 3 years of the pandemic compared to the prepandemic cohort.

**Results:**

There was a reduction in both elective and emergency IBD operations during the pandemic. These trends were not significant. After matching, the risks of returning to theaters and hospital readmission were comparable across the 3 years of the pandemic. In the first and second years of the pandemic, elective patients were at a greater risk of mortality (risk ratio [RR], 2; 95% confidence interval [CI], 1.160–3.448 and RR, 1.778; 95% CI, 1.003–3.150, respectively) and the emergency cohort had a higher risk of critical care admission (RR, 1.759; 95% CI, 1.126–2.747 and RR, 1.742; 95% CI, 1.131–2.682, respectively).

**Conclusion:**

Our study highlights the impact of the COVID‐19 pandemic on the management of IBD patients undergoing surgery. These results provide insights into the management of IBD surgery during times of crisis and can help guide decision‐making and resource allocation for IBD patients requiring surgical intervention.

## Key points

- The delivery of healthcare has been disrupted by the pandemic. Elective inflammatory bowel disease (IBD) operations were postponed to conserve healthcare resources and to reduce the spread of coronavirus disease 2019.
- There was a nonsignificant reduction in both elective and emergency IBD surgeries during the pandemic. However, elective patients in the first and second years of the pandemic faced higher mortality risks and the emergency cohort had increased critical care admissions.
- This study underscores the pandemic's impact on IBD surgery management, advocating preparedness for heightened risks, especially in elective surgery, necessitating extra resources and precautions. These insights aid decision‐making and resource allocation during crises.

## 1 INTRODUCTION

Coronavirus disease 2019 (COVID‐19), caused by severe acute respiratory syndrome coronavirus 2 (SARS‐CoV‐2), was first reported in December 2019, and has rapidly spread throughout the world leading to a global pandemic.^1^ The pandemic has affected healthcare systems worldwide, leading to changes in the delivery of healthcare services including surgical care.^2^, ^3^ Elective surgeries, including those for inflammatory bowel disease (IBD) patients, were postponed or canceled to free up resources for the management of COVID‐19 patients.^4^, ^5^ Additionally, concerns regarding the risk of COVID‐19 transmission during surgery and postoperative recovery have influenced the surgical decision‐making for IBD patients.^6^

IBD is a chronic inflammatory disorder of the gastrointestinal tract that encompasses Crohn's disease and ulcerative colitis. The management of IBD often requires surgical intervention. Surgery may be indicated for symptoms refractory to medical therapy and to manage complications of severe disease activity, such as intestinal obstruction, perforation, fistulae, and abscess formation.^7^ There are challenges to delivering safe care for IBD patients during the pandemic.^8^ IBD patients frequently require pharmacological treatment with immunosuppressant medications that can increase the risk of severe acute respiratory syndrome induced by SARS‐CoV‐2.^9^ Furthermore, elective operations were deferred to mitigate their risk of exposure to coronavirus and to minimize the use of necessary healthcare resources, such as personal protective equipment and beds in the critical care unit.

Several organizations have issued guidance on the surgical management of IBD patients during the COVID‐19 pandemic. The European Crohn's and Colitis Organisation has recommended that elective surgeries for IBD patients should be deferred if possible, and if not, should be performed in centers with adequate resources to manage COVID‐19 patients.^10^ The British Society of Gastroenterology recommended postponing elective operations and surveillance endoscopies for IBD patients to preserve healthcare resources and to prevent the spread of COVID‐19.^11^, ^12^

Delays in elective operations because of the COVID‐19 pandemic have been associated with adverse postoperative outcomes amongst cancer patients.^13^, ^14^ However, there is limited literature available on the implications of the pandemic on IBD surgical outcomes. IBD patients may be at a higher risk of postoperative complications and disease progression if their surgeries are delayed.^15^ The aim of this study is to describe the trends and outcomes of surgery in IBD patients during the COVID‐19 pandemic, from March 01, 2019 to February 28, 2023, using data from the TriNetX research network. A propensity matched analysis will be undertaken to compare adverse postoperative outcomes of IBD surgery before and during the pandemic. This study will provide valuable insights to guide future decision‐making in managing IBD patients during healthcare crises.

## 2 MATERIALS AND METHODS

### 2.1 Data source

This retrospective study used data from the TriNetX research network, a global health research network that collects and analyses electronic health records from multiple healthcare systems. The data used in this study was collected on May 23, 2023 from the TriNetX platform. TriNetX is a federated database aggregating longitudinal electronic health records of 250 million patients from over 100 healthcare organizations with real‐time updates. Data available from this network includes: (1) patient demographics, (2) diagnoses using International Classification of Diseases‐10 (ICD‐10) codes, and (3) operations using Current Procedural Terminology (CPT) codes. Data on patients is contributed from participating healthcare organizations in the network, which includes hospitals, primary care, and specialist providers. This study used only deidentified data and routinely collected data. The data extracted included patient demographics, IBD subtype, surgical procedures performed, co‐morbidities, postoperative outcomes, and the status of the surgery (elective or emergency).

### 2.2 Study population

All adult patients (age ≥ 18 years) with IBD who had undergone a surgical procedure during the COVID‐19 pandemic (March 1, 2020 to February 28, 2023) and in the preceding 12 months of the pandemic (March 1, 2019 to February 28, 2020) were included in the study. We queried the TriNetX platform to identify our cohort using a combination of ICD‐10 and CPT codes. The primary cohort was defined as patients with (1) either a diagnosis code of Crohn's disease (ICD‐10 code K50) or ulcerative colitis (ICD‐10 code K51) and (2) have had major abdominal operation for IBD (see Supporting Information: Table 1 for list of CPT codes). We excluded patients with a diagnosis of neoplasms (ICD‐10 code C00‐D49).

### 2.3 Covariates

Data including demographic co‐variates and comorbidities that could adversely affect postoperative outcomes were extracted from the database. Demographic covariates were age, gender, and ethnicity. The comorbities included: body mass index (BMI) 30‐39 (ICD‐10 code Z68.3), BMI ≥ 40 (ICD‐10 code Z68.3), diabetes mellitus (ICD‐10 code E08‐E13), hypertensive diseases (ICD‐10 code I10‐I16), ischemic heart diseases (ICD‐10 code I20‐I25), chronic kidney diseases (ICD‐10 code N18), nicotine dependence (ICD‐10 code F17), and chronic lower respiratory diseases (ICD‐10 code J40‐J47). Nicotine dependence is a condition characterized by an individual's compulsive need to use nicotine‐containing products, such as cigarettes, e‐cigarettes, cigars, or chewing tobacco.

### 2.4 Study periods

The study period (March 1, 2019 to February 28, 2023) was divided into three phases of interest: the first, second, and third years of the COVID‐19 pandemic. Each year of the pandemic was compared to the prepandemic cohort (March 1, 2019 to February 29, 2020), which represented the control.

The first year of the pandemic (March 1, 2020 to February 28, 2021) was from the time the World Health Organisation declared COVID‐19 to be a pandemic, which was in March 2020.^1^ During this time, countries around the world introduced social measures to control the spread of coronavirus. Healthcare systems were under significant pressure to preserve healthcare resources to treat COVID‐19 patients. During the second year of the pandemic (March 1, 2021 to February 28, 2022), new COVID‐19 variants emerged, and countries continued to implement social restrictions. Healthcare providers sought to restart elective operations by introducing COVID‐19‐free areas and surgical pathways to reduce patients' exposure to coronavirus.^16^ During the third year of the pandemic (March 1, 2022 to February 28, 2023), many countries began to ease restrictions as COVID‐19 cases, hospitalizations and deaths declined and vaccination rates increased.^17^

### 2.5 Study outcomes

The primary outcome was the incidence of elective (nonurgent) and emergency (urgent) operations during the pandemic compared to the prepandemic period. The secondary outcomes were adverse postoperative outcomes within 30 days of IBD surgery. After matching of the cohorts, the risks of adverse postoperative outcomes within 30 days of IBD surgery during each year of the pandemic were compared to the prepandemic cohort. Outcomes were identified with ICD‐10 and CPT codes: return to theaters, critical care admission, mortality, and hospital readmission (see Supporting Information: Table 2).

### 2.6 Data analysis

Statistical analyses were carried out using the integrated functions of the TriNetX platform. Monthly incidences of elective and emergency operations were assessed to examine trends before and during the pandemic, including mean values, standard deviations, and percentage changes. The volume of surgeries performed during the pandemic years was compared to prepandemic levels using Student's *t*‐test.

The baseline characteristics of the prepandemic and pandemic year groups were described in terms of means, standard deviations, counts, and percentages. To address potential baseline covariate imbalances, a 1:1 propensity score matching was conducted. TriNetX's built‐in function facilitated matching based on age at index, gender, ethnicity, and comorbidities such as obesity, diabetes mellitus, hypertension, ischemic heart disease, chronic kidney disease, nicotine dependence, and chronic lower respiratory diseases. The balance of baseline characteristics in the propensity score‐matched populations was assessed using the standardized difference, with values less than 0.1 considered indicative of minimal difference. A significance threshold of *p* < 0.05 was used for statistical testing.

After propensity score matching, logistic regression was used to calculate risk ratios (RRs) with 95% confidence intervals (CIs) for 30‐day postoperative outcomes. Subgroup analyses were conducted to compare elective surgeries from the prepandemic year with those during the pandemic years, as well as emergency surgeries before and during the pandemic. RRs with corresponding 95% CIs were calculated for all comparisons within the elective and emergency cohorts. Statistical analysis was performed using the TriNetX platform, with additional support from Microsoft Excel.

### 2.7 Ethical considerations

Informed consent was not obtained from patients as the study was conducted retrospectively using deidentified data. The data reviewed is a secondary analysis of existing data and does not involve intervention or interaction with human subjects. TriNetX only contains deidentified data as defined in Section §164.514(b)(1) of the HIPAA Privacy Rule, therefore ethical approval was not required. There was strict adherence to research governance guidelines throughout this study. This study was carried out under the Code of Ethics of the Word Medical Association (Declaration of Helsinki).

## 3 RESULTS

### 3.1 Trends in IBD surgery during the COVID‐19 pandemic

**Figure 1:**
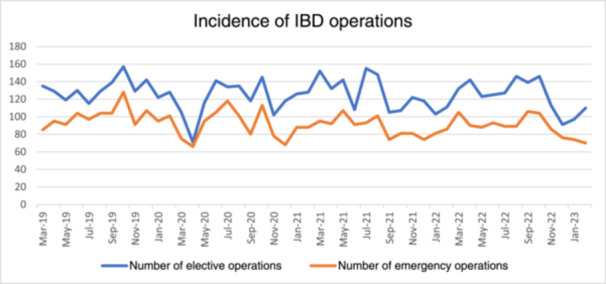
Trends in elective and emergency inflammatory bowel disease (IBD) surgical procedures before and during the coronavirus disease 2019 (COVID‐19) pandemic.

A total of 10,400 IBD operations were identified during the study period (March 1, 2019 to February 28, 2023). These procedures were performed in 39 healthcare organizations across 14 countries in Europe, Southeast Asia, North, and South America. In the year preceding the COVID‐19 pandemic, a total of 2766 surgeries were conducted, with 1574 (56.9%) classified as elective and 1192 (43.1%) as emergency procedures. During the first year of the pandemic, the number of IBD surgeries performed decreased to 2514, with 1439 (57.2%) being elective and 1075 (42.8%) emergency. In the second year, 2559 surgeries were performed, with elective surgeries accounting for 1503 (58.7%), and emergency surgeries for 1056 (41.3%). By the third year, 2561 surgeries were conducted, with 1491 (58.2%) classified as elective and 1070 (41.8%) as emergency procedures. The monthly incidence of elective and emergency operations performed during the study period (March 1, 2020 to February 28, 2023) is shown in Figure 1.

There was a reduction in both elective and emergency IBD surgeries during the pandemic compared with the prepandemic period (Table 1). The mean number of elective operations per month was 131.2 (±11.2) before the COVID‐19 pandemic, and 119.9 (±20.4), 125.3 (±19.6), and 124.3 (±18.4) in the first, second and third years of the pandemic, respectively. The first year of the pandemic observed the largest percent decline in elective procedures (−11.25); however, this was not significant (*p* = 0.06). The month with the lowest number of elective operations was in April 2020 (*n* = 71), compared to the same month in the previous year (*n* = 129). For emergency IBD admissions, the mean number of operations per month was 99.3 (±11.6) before the COVID‐19 pandemic, and 89.6 (±17.1), 88 (±10.3), and 89.2 (±11.9) in the first, second and third years of the pandemic, respectively. The second year of the pandemic observed the largest percent decline in emergency procedures (−11.3); however, this was not significant (*p* = 0.7).

**Table 1:** Trends in elective and emergency IBD surgical procedures during the pandemic compared to prepandemic levels. The figures represent the average number of operations conducted per month.

	Prepandemic	First year of COVID			Second year of COVID			Third year of COVID		
	Mean (SD)	Mean (SD)	Percent change	*p*‐Value	Mean (SD)	Percent change	*p*‐Value	Mean (SD)	Percent change	*p*‐Value
Elective	131.2 (11.2)	119.9 (20.4)	−11.25	0.06	125.3 (19.6)	−5.9	0.08	124.3 (18.4)	−6.9	0.1
Emergency	99.3 (11.6)	89.6 (17.1)	−9.750	0.2	88.0 (10.3)	−11.3	0.7	89.2 (11.9)	−10.2	0.9

Abbreviations: COVID, coronavirus disease 2019; IBD, inflammatory bowel disease; SD, standard deviation.

### 3.2 Study population

Tables 2, 3, 4 provides information on the baseline characteristics of the studied cohorts across the 3 years of the pandemic compared to the prepandemic cohort. The tables include demographics (age, gender, and ethnicity), as well as comorbidities (BMI, diabetes mellitus, hypertension, ischemic heart disease, chronic kidney disease, nicotine dependence, and chronic lower respiratory diseases).

**Table 2:** Baseline characteristics of patients operated on in the prepandemic period and in the first year of COVID‐19.

Variable	Before matching				After matching			
	Prepandemic (*n* = 2414)	First year of COVID (*n* = 2156)	*p*‐Value	Standard difference	Prepandemic (*n* = 2089)	First year of COVID (*n* = 2089)	*p*‐Value	Standard difference
Demographics								
Age; mean (SD)	42.0 (19.4)	44.0 (19.5)	0.001	0.102	43.5 (19.4)	43.3 (19.2)	0.9	0.003
Gender; *n* (%)								
Male	1226 (50.8)	1018 (47.2)	0.02	0.071	994 (47.6)	1002 (48.0)	0.8	0.008
Female	1188 (49.2)	1138 (52.8)	0.02	0.071	1095 (52.4)	1087 (52.0)	0.8	0.008
Ethnicity; *n* (%)								
White	1772 (73.4)	1543 (71.6)	0.2	0.041	1511 (72.3)	1510 (72.3)	>0.99	0.001
Black or African American	352 (14.6)	336 (15.6)	0.3	0.028	323 (15.5)	323 (15.5)	>0.99	<0.001
Unknown	237 (9.8)	220 (10.2)	0.7	0.013	210 (10.1)	212 (10.1)	0.9	0.003
Asian	41 (1.7)	49 (2.3)	0.2	0.041	39 (1.9)	39 (1.9)	>0.99	<0.001
Comorbidities								
Body mass index 30–39; *n* (%)	195 (8.1)	195 (9.0)	0.2	0.035	184 (8.8)	174 (8.3)	0.6	0.017
Body mass index ≥ 40; *n* (%)	72 (3.0)	72 (3.3)	0.5	0.020	65 (3.1)	69 (3.3)	0.7	0.011
Diabetes mellitus; *n* (%)	301 (12.5)	305 (14.1)	0.1	0.049	270 (12.9)	281 (13.5)	0.6	0.016
Hypertension; *n* (%)	721 (29.9)	714 (33.1)	0.02	0.070	660 (31.6)	671 (32.1)	0.7	0.011
Ischemic heart diseases; *n* (%)	254 (10.5)	280 (13.0)	0.01	0.077	242 (11.6)	248 (11.9)	0.8	0.009
Chronic kidney diseases; *n* (%)	212 (8.8)	224 (10.4)	0.07	0.055	201 (9.6)	208 (10.0)	0.7	0.011
Nicotine dependence; *n* (%)	498 (20.6)	460 (21.3)	0.6	0.017	441 (21.1)	444 (21.3)	0.6	0.016
Chronic lower respiratory diseases; *n* (%)	474 (19.6)	457 (21.2)	0.2	0.039	417 (20.0)	438 (21.0)	0.4	0.025

Abbreviations: COVID, coronavirus disease 2019; SD, standard deviation.

**Table 3:** Baseline characteristics of patients operated on in the prepandemic period and in the second year of COVID‐19.

Variable	Before matching				After matching			
	Prepandemic (*n* = 2414)	Second year of COVID (*n* = 2227)	*p*‐Value	Standard difference	Prepandemic (*n* = 2068)	Second year of COVID (*n* = 2068)	*p*‐Value	Standard difference
Demographics								
Age; mean (SD)	42.0 (19.4)	43.9 (19.3)	0.001	0.098	43.5 (19.4)	42.9 (19.1)	0.3	0.031
Gender; *n* (%)								
Male	1226 (50.8)	1048 (47.1)	0.01	0.075	1006 (48.6)	1000 (48,4)	0.9	0.006
Female	1188 (49.2)	1179 (52.9)	0.01	0.075	1062 (51.4)	1068 (51.6)	0.9	0.006
Ethnicity; *n* (%)								
White	1772 (73.4)	1584 (71.1)	0.08	0.051	1495 (72.3)	1481 (71.6)	0.6	0.015
Black or African American	352 (14.6)	337 (15.1)	0.6	0.015	301 (14.6)	317 (15.3)	0.5	0.022
Unknown	237 (9.8)	258 (11.6)	0.05	0.057	228 (11.0)	224 (10.8)	0.8	0.006
Asian	41 (1.7)	39 (1.8)	0.9	0.004	38 (1.8)	37 (1.8)	0.9	0.004
Comorbidities								
Body mass index 30–39; *n* (%)	195 (8.1)	206 (9.3)	0.2	0.042	183 (8.8)	178 (8.6)	0.8	0.009
Body mass index ≥ 40; *n* (%)	72 (3.0)	84 (3.8)	0.1	0.044	63 (3.0)	67 (3.2)	0.7	0.011
Diabetes mellitus; *n* (%)	301 (12.5)	346 (15.5)	0.003	0.088	276 (13.3)	290 (14.0)	0.5	0.020
Hypertension; *n* (%)	721 (29.9)	752 (33.8)	0.004	0.084	666 (32.2)	660 (31.9)	0.8	0.006
Ischemic heart diseases; *n* (%)	254 (10.5)	287 (12.9)	0.01	0.074	421 (11.7)	247 (11.9)	0.8	0.009
Chronic kidney diseases; *n* (%)	212 (8.8)	247 (11.1)	0.008	0.077	191 (9.2)	209 (10.1)	0.3	0.029
Nicotine dependence; *n* (%)	498 (20.6)	460 (20.7)	>0.99	0.001	417 (20.2)	422 (20.4)	0.8	0.006
Chronic lower respiratory diseases; *n* (%)	474 (19.6)	489 (22.0)	0.05	0.057	437 (21.1)	423 (20.5)	0.6	0.017

Abbreviations: COVID, coronavirus disease 2019; SD, standard deviation.

**Table 4:** Baseline characteristics of patients operated on in the prepandemic period and in the third year of COVID‐19.

Variable	Before matching				After matching			
	Prepandemic (*n* = 2414)	Third year of COVID (*n* = 2211)	*p*‐Value	Standard difference	Prepandemic (*n* = 2093)	Third year of COVID (*n* = 2093)	*p*‐Value	Standard difference
Demographics								
Age; mean (SD)	42.0 (19.4)	43.7 (19.1)	0.003	0.088	43.3 (19.4)	43.2 (19.0)	0.9	0.006
Gender; *n* (%)								
Male	1226 (50.8)	1071 (48.4)	0.1	0.047	1036 (49.5)	1026 (49.0)	0.8	0.010
Female	1188 (49.2)	1140 (51.6)	0.1	0.047	1057 (50.5)	1067 (51.0)	0.8	0.010
Ethnicity; *n* (%)								
White	1772 (73.4)	1584 (71.6)	0.2	0.040	1524 (72.8)	1521 (72.7)	0.9	0.003
Black or African American	352 (14.6)	300 (13.6)	0.3	0.029	289 (13.8)	286 (13.7)	0.9	0.004
Unknown	237 (9.8)	268 (12.1)	0.01	0.074	234 (11.2)	236 (11.3)	0.9	0.003
Asian	41 (1.7)	50 (2.3)	0.2	0.040	41 (2.0)	43 (2.1)	0.8	0.007
Comorbidities								
Body mass index 30–39; *n* (%)	195 (8.1)	225 (10.2)	0.01	0.073	186 (8.9)	181 (8.6)	0.8	0.008
Body mass index ≥ 40; *n* (%)	72 (3.0)	92 (4.2)	0.03	0.064	70 (3.3)	68 (3.2)	0.9	0.005
Diabetes mellitus; *n* (%)	301 (12.5)	350 (15.8)	0.001	0.097	294 (14.0)	298 (14.2)	0.9	0.005
Hypertension; *n* (%)	721 (29.9)	735 (33.2)	0.01	0.073	667 (31.9)	668 (31.9)	0.97	0.001
Ischemic heart diseases; *n* (%)	254 (10.5)	276 (12.5)	0.04	0.061	244 (11.7)	251 (12.0)	0.7	0.010
Chronic kidney diseases; *n* (%)	212 (8.8)	215 (9.7)	0.3	0.033	194 (9.3)	199 (9.5)	0.8	0.008
Nicotine dependence; *n* (%)	498 (20.6)	453 (20.5)	0.9	0.003	427 (20.4)	434 (20.7)	0.8	0.008
Chronic lower respiratory diseases; *n* (%)	474 (19.6)	439 (19.9)	0.9	0.006	407 (19.4)	415 (19.8)	0.8	0.010

Abbreviations: COVID, coronavirus disease 2019; SD, standard deviation.

### 3.3 Adverse outcomes within 30 days following IBD surgery during the COVID‐19 pandemic

There were no significant differences in the mean age of the cohorts throughout the study periods. There were slight differences in the prevalence of co‐morbidities between the prepandemic and pandemic groups. The mean body mass index was slightly higher amongst the pandemic cohorts. There was a marginally higher prevalence of diabetes mellitus, hypertension, ischemic heart diseases, chronic kidney diseases, and chronic lower respiratory diseases amongst the pandemic populations compared to the prepandemic group. After matching, the standard differences were less than 0.05. There were no significant differences in the baseline characteristics between the prepandemic and pandemic cohorts, suggesting that the matched cohorts were comparable.

Tables 5 and 6 show the proportion of patients and the risk of adverse outcomes following IBD surgery during the first, second, and third years of the pandemic, compared to the prepandemic cohort, after propensity score matching.

**Table 5:** Proportion and risks of adverse outcomes following elective IBD operations for each year of the pandemic compared to prepandemic controls.

Elective operations after propensity score matching						
Clinical outcomes	First year of pandemic	Prepandemic	Second year of pandemic	Prepandemic	Third year of pandemic	Prepandemic
Return to theaters						
*n* (%)	155 (13.5)	149 (13)	166 (13.8)	164 (13.7)	165 (14.1)	164 (14.0)
RR (95% CI)	1.040 (0.844–1.283)		1.012 (0.828–1.237)		1.006 (0.823–1.229)	
*p*‐Value	0.7		0.9		0.2	
Critical care admission						
*n* (%)	101 (8.8)	81 (7)	93 (7.7)	89 (7.4)	89 (7.6)	90 (7.7)
RR (95% CI)	1.247 (0.942–1.651)		1.045 (0.790–1.382)		0.989 (0.746–1.310)	
*p*‐Value	0.1		0.8		0.9	
Mortality						
*n* (%)	38 (3.3)	19 (1.7)	32 (2.7)	18 (1.5)	24 (2.0)	19 (1.6)
RR (95% CI)	2 (1.160–3.448)		1.778 (1.003–3.150)		1.263 (0.696–2.293)	
*p*‐Value	0.01		0.05		0.4	
Hospital readmission						
*n* (%)	150 (13.0)	150 (13.0)	150 (12.5)	163 (13.6)	138 (11.8)	161 (13.7)
RR (95% CI)	1 (0.810–1.235)		0.920 (0.748–1.132)		0.857 (0.693–1.060)	
*p*‐Value	>0.99		0.4		>0.99	

Abbreviations: 95% CI, 95% confidence interval; IBD, inflammatory bowel disease; RR, risk ratio.

**Table 6:** Proportion and risks of adverse outcomes following emergency IBD operations for each year of the pandemic compared to prepandemic controls.

Emergency after propensity score matching						
Clinical outcomes	First year of pandemic	Prepandemic	Second year of pandemic	Prepandemic	Third year of pandemic	Prepandemic
Return to theaters						
*n* (%)	128 (14.7)	134 (15.4)	134 (15.3)	141 (16.1)	134 (15.0)	148 (16.5)
RR (95% CI)	0.955 (0.764–1.194)		0.950 (0.765–1.181)		0.905 (0.731–1.122)	
*p*‐Value	0.7		0.2		0.2	
Critical care admission						
*n* (%)	51 (5.9)	29 (3.3)	54 (6.2)	31 (3.5)	49 (5.5)	36 (4.0)
RR (95% CI)	1.759 (1.126–2.747)		1.742 (1.131–2.682)		1.361 (0.894–2.072)	
*p*‐Value	0.01		0.01		0.4	
Mortality						
*n* (%)	13 (1.5)	10 (1.1)	15 (1.7)	10 (1.1)	14 (1.6)	10 (1.1)
RR (95% CI)	1.300 (0.573–2.949)		1.500 (0.678–3.321)		1.400 (0.625–3.135)	
*p*‐Value	0.5		0.3		0.4	
Hospital readmission						
*n* (%)	134 (15.4)	160 (18.4)	145 (16.5)	160 (18.2)	152 (17.0)	167 (18.6)
RR (95% CI)	0.838 (0.679–1.033)		0.906 (0.739–1.112)		0.910 (0.746–1.111)	
*p*‐Value	0.1		0.3		0.4	

Abbreviations: 95% CI, 95% confidence interval; IBD, inflammatory bowel disease; RR, risk ratio.

For elective operations, there were no significant differences in the risk of returning to theaters, critical care admission, and hospital readmission across the 3 years of the pandemic compared to the prepandemic group. Elective patients had a higher risk of mortality in the first and second years of the pandemic compared to the prepandemic cohort (RR, 2; 95% CI, 1.160–3.448; *p* = 0.01, and RR, 1.778; 95% CI, 1.003–3.150; *p* = 0.05, respectively). While the mortality risk difference in the first year is significant, the difference in the second year is on the threshold of conventional significance levels.

For emergency operations, the risk of returning to theaters, mortality, and hospital readmission were not significant across the 3 years of the pandemic compared to the prepandemic group. Emergency patients had a higher risk of critical care admission in the first and second years of the pandemic compared to the prepandemic cohort (RR, 1.759; 95% CI, 1.126–2.747; *p* = 0.01 and RR, 1.742, 95% CI 1.131–2.682; *p* = 0.01, respectively). The risk differences in critical care admission during these 2 years are significant.

## 4 DISCUSSION

Our study aimed to describe the trends and outcomes of surgery in IBD patients during the COVID‐19 pandemic, using data from the TriNetX research network. Our findings have shown that the pandemic has affected the incidence of both elective and emergency IBD surgeries. The total number of operations performed during the pandemic was comparably lower to the prepandemic period. The reduction in elective surgeries during the pandemic period could be attributed to several factors. Elective, or nonurgent, surgeries were deferred to reduce the risk of exposure to SARS‐CoV‐2 in healthcare settings and to prioritize healthcare resources towards patients affected by COVID‐19. In addition, patients may have chosen to delay nonurgent operations due to concerns of contracting SARS‐CoV‐2 in hospitals.^18^ Despite a reduction in elective IBD operations, we did not observe a surge in emergency cases throughout the pandemic. IBD patients seeking emergency treatment declined in the United Kingdom at the start of the pandemic.^19^ The reduction in emergency operations suggests that urgent surgical IBD procedures were still being performed as needed during the pandemic; however, patients may have attempted to avoid emergency hospital attendances.^20^, ^21^

It is hypothesized that the reduction in elective surgeries could result in delayed treatment and worse outcomes for patients.^22^, ^23^, ^24^ Contrary to this, a single‐center study conducted in Italy reported that the mortality rate and postoperative complications were similar in IBD patients who underwent surgery before and during the pandemic.^25^ In our study, we observed a similar risk of returning to theaters and hospital readmissions amongst patients that had surgery during the pandemic compared to the prepandemic period. However, patients who had an elective IBD operation, in the first 2 years of the pandemic, were observed to have a higher risk of mortality, and those who had an emergency procedure were found to have a higher risk of critical care admission.

The reasons for these risk differences are likely to be multi‐factorial. First, the social restrictions and the diversion of healthcare resources may have delayed referrals and access to diagnostic and treatment for IBD patients.^26^ Patients who experience a delayed diagnosis of IBD have associated worse treatment outcomes and poorer quality of life. They are also at a higher risk of developing IBD‐related complications.^27^ Additionally, the emergence of new, highly transmissible variants of COVID‐19 has increased the chances of IBD patients contracting coronavirus. If infected, those on steroid therapy are more likely to experience severe COVID‐19 and may require admission to a critical care unit, compared to non‐IBD patients.^28^, ^29^ The PREPARE‐IBD study observed notable changes in the medical management of IBD in the UK during the pandemic.^30^ There was a significant reduction in the use of systemic corticosteroid therapy among patients. Before the pandemic, systemic corticosteroids were commonly prescribed for managing IBD flares. However, during the pandemic, their prescription declined, likely reflecting concerns among IBD physicians that these medications could increase the risk of severe COVID‐19 outcomes due to their immunosuppressive effects. A global multi‐center study revealed that the presence of COVID‐19 during surgery can increase the risk of postoperative pulmonary complications, critical care unit admission, and mortality.^31^, ^32^ In response to the initial wave of COVID‐19, dedicated surgical pathways were established to ensure the safe resumption of elective surgeries while mitigating their risk to COVID‐19.^16^ Patients following these pathways had lower rates of postoperative SARS‐CoV‐2 infection, and pulmonary complications.

It is important to note that the outcomes of surgery during the pandemic may also depend on other factors, such as the availability of critical care resources, and patient factors such as disease severity, prior IBD drug therapies, co‐morbidities, and COVID‐19 vaccine status.^33^, ^34^ Several studies have found a correlation between severe COVID‐19 infection and certain factors, such as advancing age, the presence of co‐morbidities and the concurrent use of steroid therapy.^35^, ^36^, ^37^ A study using data from the TriNetX platform had identified that COVID‐19‐vaccinated IBD patients were at a lower risk of severe adverse COVID‐19 outcomes compared with unvaccinated IBD patients.^38^ Furthermore, they have reported that patients who had received three doses of the vaccine were at a reduced risk of hospitalization compared to those who had two doses. In view of this, IBD surgery can be safely performed in a selected patient population despite the challenges posed by the pandemic. We recommend that individuals with IBD receive the COVID‐19 vaccination and healthcare organizations should consider dedicated surgical pathways for elective IBD patients to effectively reduce the risks associated with coronavirus infection.

Findings from this study provide valuable insights into the impact of the COVID‐19 pandemic on the outcomes of IBD surgery patients and can be used to inform clinical practice, policy decisions and resource allocation in unprecedented healthcare settings. Surgical decision‐making for IBD patients should consider the risk of COVID‐19 transmission, the urgency of surgery, and the availability of resources.^39^ Overall, these findings underscore the importance of ensuring that healthcare systems are prepared to maintain the quality of care for patients with IBD and other chronic conditions during pandemics and other crises. Further research is needed to explore the impact of the pandemic on other aspects of IBD care, such as medication adherence and disease progression. Moreover, studies are needed to assess the longer‐term impact of delayed or postponed surgeries on patient outcomes.

## 5 LIMITATIONS

This study has several limitations. First, it is a retrospective study, and therefore, the findings are subject to selection bias and confounding variables. The study did not evaluate the impact of medication usage, specifically IBD drug therapies, or disease severity, all of which can have a significant impact on postoperative outcomes. Second, the study relied on electronic health records. There may have been incomplete or inaccurate data, such as incorrect ICD‐10 or CPT codes assigned to patients. Third, the study did not specially consider the impact of infection with SARS‐CoV‐2 amongst IBD surgical patients. The cause of death is undisclosed, it is unclear whether patients died because of COVID‐19 infection or a complication from surgery. Four, separating the pandemic into distinct stages or “waves” is challenging because each country experienced different lockdown periods, vaccination timelines, and healthcare system pressures. The chosen time periods were arbitrary, designed to fit into 12‐month segments. It is important to acknowledge that the pandemic's progression was dynamic, with each country implementing unique national policies to manage COVID‐19. Consequently, the impact of coronavirus varied across countries at different times, with what constitutes a “wave” in one country potentially occurring at a different time in another.

Fifth, the study was conducted using data from the TriNetX research network. TriNetX is a live network, which provides real‐time updates. Patient counts can vary daily; new healthcare organizations are being put online and some healthcare organizations may be offline due to maintenance and while data mappings are modified. Finally, the specific types of healthcare institutions, as well as the countries involved, are not known due to the federated and aggregated nature of the TriNetX database. TriNetX includes data from over 120 healthcare organizations across 19 countries globally. Because the data is deidentified, it is not possible to determine the originating country or hospital, ensuring patient anonymity. Consequently, it is challenging to assess how generalizable or applicable the conclusions are to specific healthcare settings.

## 6 CONCLUSION

This study provides important insights into the trends and outcomes of IBD surgery during the COVID‐19 pandemic. Using data extracted from TriNetX platform, our study has observed a decline in both the number of elective and emergency IBD surgeries during the pandemic. After matching, the risk of returning to theaters and hospital readmissions were comparable before and during the pandemic. In the first and second years of the pandemic, elective patients were at a greater risk of mortality and the emergency cohort had a higher risk of critical care admission. These findings may reflect changes in healthcare resources and staffing during the pandemic, as well as variations in the severity of COVID‐19 outbreaks over time. Future studies should focus on identifying the factors contributing to these outcomes and developing strategies to mitigate the impact of future healthcare crises on the care of IBD patients.

## AUTHOR CONTRIBUTIONS

**Fiona Wu**: Writing—original draft; writing—review and editing; formal analysis; data curation. **Gema H. Ibarburu**: Methodology; formal analysis; software; data curation; resources; project administration. **Caris Grimes**: Conceptualization; writing—review and editing; validation; supervision; resources.

## CONFLICT OF INTEREST STATEMENT

The authors declare no conflict of interest.

## TRANSPARENCY STATEMENT

The lead author Caris Grimes affirms that this manuscript is an honest, accurate, and transparent account of the study being reported; that no important aspects of the study have been omitted; and that any discrepancies from the study as planned (and, if relevant, registered) have been explained.

## Supporting information

Supporting information.

## Data Availability

The data that support the findings of this study are available from the corresponding author upon reasonable request. Data can be made available on request. The lead author had full access to all of the data in this study and took complete responsibility for the integrity of the data and the accuracy of the data analysis.
