# Supplementary material for: The trends and outcomes of inflammatory bowel disease surgery during the COVID‐19 pandemic: A retrospective propensity score‐matched analysis from a multi‐institutional research network
Source: Health Sci Rep. 2024 Sep 29;7(10):e70107. doi: 10.1002/hsr2.70107 (PMC11439741; doi:10.1002/hsr2.70107)
Supplement: Supplementary file 1 — Supporting information. [file HSR2-7-e70107-s001.docx]

***Supplemental tables***

| **ICD-10/CPT code** | **Definition** |
| --- | --- |
| CPT 44615 | Intestinal stricturoplasty (enterotomy and enterorrhaphy) with or without dilation, for intestinal obstruction |
| ICD-10 PCS 0DT8 | Gastrointestinal System / Resection / Small Intestine |
| ICD-10 PCS 0DTE | Gastrointestinal System / Resection / Large Intestine |
| ICD-10 PCS 0DTP | Gastrointestinal System / Resection / Rectum |
| CPT 1007455 | Colectomy, partial |
| CPT 1007463 | Colectomy, total, abdominal, without proctectomy |
| CPT 1007468 | Colectomy, total, abdominal, with proctectomy |
| ICD-10 PCS 0D1B0ZQ | Bypass Ileum to Anus, Open Approach |
| CPT 1007422 | Surgical Procedures on the Intestines (Except Rectum) |
| CPT 1007591 | Surgical Procedures on the Colon and Rectum |

**Supplemental table 1:** ICD-10 and CPT codes of IBD operations.

| **Outcome** | **ICD-10/CPT code** | **Definition** |
| --- | --- | --- |
| **Return to theatres** | CPT 1007422 | Surgical Procedures on the Intestines (Except Rectum) |
|  | CPT 1007591 | Surgical Procedures on the Colon and Rectum |
|  | CPT 1007695 | Surgical Procedures on the Anus |
|  | CPT 1007952 | Surgical Procedures on the Abdomen, Peritoneum, and Omentum |
| **Critical care admission** | CPT 1013729 | Critical Care Services |
| **Mortality** | ICD-10 CM R99 | Ill-defined and unknown cause of mortality |
| **Hospital**  **re-admission** | CPT 1013711 | Emergency Department Services |

**Supplemental table 2:** ICD-10 and CPT codes of adverse post-operative outcomes.
